# Novel Micro/Nanomaterial-Based Drug Delivery Systems for Theranostic Applications

**DOI:** 10.3390/pharmaceutics18101228

**Published:** 2026-09-28

**Authors:** Pedro Brandão, Sofia O. D. Duarte, Pedro Fonte

**Affiliations:** 1 iBB-Institute for Bioengineering and Biosciences, Department of Bioengineering, Instituto Superior Técnico, University of Lisboa, 1049-001 Lisbon, Portugal; pbrandao@egasmoniz.edu.pt (P.B.); sofia.duarte@tecnico.ulisboa.pt (S.O.D.D.); 2 Associate Laboratory i4HB–Institute for Health and Bio-Economy, Instituto Superior Técnico, University of Lisboa, Av. Rovisco Pais, 1049-001 Lisbon, Portugal; 3 Egas Moniz Center for Interdisciplinary Research (CiiEM), Egas Moniz School of Health & Science, 2829-511 Almada, Portugal; 4 Coimbra Chemistry Centre–Institute of Molecular Sciences (CQC-IMS), Department of Chemistry, University of Coimbra, Rua Larga, 3004-535 Coimbra, Portugal; 5 Centro de Ciências do Mar do Algarve (CCMAR/CIMAR LA), Campus de Gambelas, Universidade do Algarve, 8005-139 Faro, Portugal; 6 Department of Chemistry and Pharmacy, Faculty of Sciences and Technology, University of Algarve, Gambelas Campus, 8005-139 Faro, Portugal

## 1. Introduction

The field of modern medicine is currently undergoing a paradigm shift, driven by the need to overcome the inherent limitations of conventional therapeutic and diagnostic interventions. Traditional drug delivery methods often suffer from sub-optimal bioavailability, non-specific distribution and systemic toxicity, which constrain therapeutic dosages and compromise patient outcomes [1,2]. In response, the emergence of theranostics, an integrated platform combining therapeutic and diagnostic functions, represents a significant advancement toward precision medicine [3]. By using micro- and nanomaterials, researchers can now design systems that not only deliver therapeutic payloads with high specificity but also provide real-time monitoring of disease progression and treatment efficacy [4,5]. The Special Issue of *Pharmaceutics*, titled “Novel Micro/Nanomaterials Based Drug Delivery Systems for Theranostic Applications,” brings together a diverse collection of reviews and original research that highlight the state of the art in this rapidly evolving field.

## 2. Overview of the Contributions to the Special Issue

The contributions to this Special Issue include comprehensive reviews that establish the design principles and versatile applications of new nanoplatforms. Tian et al. provides a foundational overview of microscale and nanoscale materials, categorizing them into organic, inorganic, and composite systems. Their work details how these materials can be tailored for oncology, cardiovascular disorders, and infectious diseases through functionalization with targeting ligands and stimuli-responsive mechanisms. They emphasize that the convergence of imaging modalities like magnetic resonance imaging (MRI), computed tomography (CT), and fluorescence with therapeutic delivery allows for a personalized approach to chronic disease management [6].

In another review published in the Special Issue, Almatroudi et al. focuses specifically on the multifaceted potential of silver nanoparticles (AgNPs). The review explores the “alchemical” domains of AgNP synthesis, contrasting traditional chemical and physical methods with the increasingly popular green approaches utilizing plant extracts and microorganisms. Beyond their well-known broad-spectrum antibacterial, antifungal, and antiviral properties, AgNPs are highlighted for their emerging roles as innovative drug carriers and anticancer agents capable of disrupting tumor growth with high precision [7].

The original research articles in the Special Issue further demonstrate the practical application and latest developments in this field. In the realm of inorganic nanomaterials for oncology, Martins et al. investigated the synergistic potential of combining gold nanoparticles (AuNPs) with ionizing radiation to treat pancreatic cancer. Their study demonstrates that functionalized AuNPs, in particular those coated with hyaluronic and oleic acids or bombesin peptides, can act as potent radiosensitizers in vitro, significantly enhancing cytotoxicity against BxPC-3 tumor cells when compared to radiotherapy alone. This work underscores the importance of surface modification in improving the targeting and therapeutic ratio of metallic nanoparticles [8].

Advancing the development of sustainable diagnostic tools, Rodrigues et al. report on the synthesis of carbon dots (Cdots) derived from Ceiba speciosa biomass for uterine cancer bioimaging. These Cdots, prepared via a low-cost ecofriendly process, when loaded into carboxymethylcellulose-based hydrogels, exhibit intrinsic photoluminescence and high biocompatibility. This research illustrates how sustainable nanomaterials can be integrated into polymeric matrices to create effective, non-invasive diagnostic nanoprobes for early-stage neoplastic detection in women’s health [9].

The Special Issue also explores smart stimuli-responsive systems, as demonstrated by Duan et al., who developed a glutathione (GSH)-responsive paclitaxel (PTX) prodrug. By using an endogenous molecule, oxidized L-glutathione (GSSG), the researchers engineered highly stable micelles that are selectively disrupted in the high-GSH environment of tumor cells. This approach not only ensures a reliable drug release rate but also significantly improves the biocompatibility of chemotherapy, as evidenced by reduced hemolysis compared to traditional albumin-bound formulations [10].

Shaban et al. expanded the horizons of nanotheranostics by exploring the delivery of natural bioactive compound (hesperidin) for organ protection, namely, to prevent acute paracetamol-induced hepatotoxicity in an in vivo model (Wistar rats). They used MgAl-layered double hydroxide nanocomposite loaded with hesperidin as the therapeutic approach. Their findings suggest that nanoformulations can significantly enhance the efficacy of natural products by controlling biotransformation, oxidative stress, and inflammatory pathways, thereby offering a potent strategy for managing drug-induced liver injury [11].

## 3. Current Knowledge Gaps and Translational Challenges

Despite substantial preclinical progress, the clinical translation of theranostic micro/nanoscale systems remains hindered by several critical obstacles. A primary concern is the long-term biocompatibility and systemic toxicity of inorganic and metal-based nanoparticles, such as gold and silver, which risk accumulating in the liver, spleen, and kidneys, potentially leading to chronic inflammation or oxidative stress [12,13]. Furthermore, the complexity of engineering “all-in-one” platforms often results in a “conflict of requirements,” where optimizing a nanocarrier for high-resolution imaging may compromise its drug-loading capacity or structural stability [14].

Biological heterogeneity also poses a significant challenge to the predictability of pharmacokinetics and biodistribution. In oncology, for example, while many systems rely on the enhanced permeability and retention (EPR) effect for passive tumor targeting, this phenomenon is notoriously inconsistent across different human tumor types and patient populations [15,16]. Additionally, the premature leakage of diagnostic or therapeutic agents before reaching the target site can cause off-target toxicities in healthy tissues [17]. From a manufacturing perspective, the transition from laboratory-scale synthesis to large-scale industrial production is complicated by high costs, batch-to-batch variability, and the lack of standardized protocols for quality control and regulatory safety assessments [14].

## 4. Future Research Directions

To bridge the gap between benchtop innovation and bedside application, future research should focus on process sustainability, technological innovation, and personalized medicine. Sustainable synthesis of nanotheranostic tools is not only cost-effective, but also capable of producing highly biocompatible materials with reduced environmental footprints. The arising of fields such as nanoinformatics and the opportunity to integrate artificial intelligence can offer powerful tools for the rational design of nanoparticles. Machine learning algorithms can analyze vast datasets to predict nanoparticle behavior in complex biological environments, thereby accelerating the screening of optimal formulations and minimizing experimental trial-and-error. In the field of personalized medicine, the emergence of multimodal synergistic therapies, which combine multiple therapeutic modalities, as well as advances in genomic technologies that enable personalized molecular profiling, will generate customizable nanotheranostic tools that will facilitate individualized treatment plans, while maximizing efficacy and overcoming drug resistance and adverse effects.

## 5. Conclusions

The contributions to this Special Issue underscore the transformative potential of micro/nanomaterials in redefining the landscape of modern medicine. By merging therapy and diagnosis into unified theranostic platforms, these novel systems offer a roadmap to overcome the historical barriers of non-specific drug delivery and late-stage disease detection. While challenges regarding toxicity, scalability, and regulatory approval persist, the ongoing advancements in stimuli-responsive materials and biomimetic engineering provide a robust foundation for safer and more effective interventions. Realizing the full promise of nanotheranostics will require sustained interdisciplinary collaboration between material scientists, pharmacists, and clinicians to ensure these life-changing technologies reach the global patient population.
